# Interruptions of activities experienced by nursing professionals in an intensive care unit**^1^**

**DOI:** 10.1590/1518-8345.0997.2802

**Published:** 2016-09-09

**Authors:** Daniele de Oliveira Prates, Ana Elisa Bauer de Camargo Silva

**Affiliations:** 2 RN, Secretaria Municipal de Saúde, Goiânia, GO, Brazil.; 3 Adjunct Professor, Faculdade de Enfermagem, Universidade Federal de Goiás, Goiânia, GO, Brazil.

**Keywords:** Patient Safety, Nursing Care, Intensive Care Units, Observation

## Abstract

**Objective:**

to analyze the interruptions experienced by nursing professionals while undertaking care activities.

**Method:**

an observational study undertaken in two intensive care units. Two nurses observed 33 nursing professionals for three hours. The data were recorded in real time, using a semistructured instrument.

**Results:**

after 99 hours of observation of 739 activities, it was identified that 46.82% were interrupted, resulting in 7.85 interruptions per hour. On average, the interruptions compromised 9.42% of the nursing professionals' worktime. The activities geared towards indirect care of the patient suffered the highest number of interruptions (56.65%), with the nursing records being the activity interrupted most. The principal source of the interruptions was external, coming from the health professionals (51%), and the main causes were those related to the patients (34.70%) and to interpersonal communication (26.47%).

**Conclusion:**

the activity of nursing suffers a high number of interruptions, mainly caused by the health professionals themselves, indicating that the work environment needs to undergo interventions aiming to reduce the risk of compromising of the professional's performance and to increase the patients' safety.

## Introduction

Patient safety has been much discussed over recent decades, bearing in mind that many users of the health service have suffered adverse events as a result of errors which took place in the care. The Institute of Medicine's publication, "To Err is Human: Building a Safer Health System" identified that avoidable adverse events are the fourth most important cause of death in the United States of America (USA)^1^. 

The interruptions experienced by the health professionals while undertaking their care activities were indicated as possible factors for errors, establishing a causal relationship between patient safety and the occurrence of interruptions; since 2008, this issue has been strongly studied by health researchers^2^ ^-^ ^3^. 

The interruptions - acts which disrupt or suspend an activity - are derived from external events originating from people or from the sounds of equipment, such as telephones and alarms, or from self-interruption^4^. The interruptions contribute to distracting human attention, possibly resulting in disrupting or interrupting the activity which is being undertaken, even if temporarily, reducing time for reflection and ability to think^4^ ^-^ ^5^. After experiencing the interruption, the professional runs the risk of omitting or repeating some steps, or it may even happen that the entire task may be repeated, which may have disastrous effects^6^. 

In this context, the interruptions must be the focus of attention in health institutions, which are considered complex environments, as they may be harmful to the patients' safety^7^. 

In one investigation undertaken in two teaching hospitals in Australia, the nurses who were interrupted presented higher chances of committing errors^8^. In the USA, one study indicated that the interruptions and distractions were responsible for more than half of the events reported (59.6%) associated with drug errors^5^. Evidence also indicates an association between interruptions and distractions of the surgical team and an increase in mortality^4^.

The Intensive Care Unit (ICU) deserves to be emphasized, as it presents considerable challenges in relation to patient safety, considering that highly complex processes are undertaken in this unit. The nursing care in this unit requires much attention from the professionals who frequently need to take fast and risky decisions, in addition to undertaking a high number of invasive interventions and using a variety of devices, various high-alert medications and new therapeutic technologies, with studies indicating the high incidence of adverse events^9^ ^-^ ^10^.

Considering the need to identify situations of risk existing in nursing's work environment, which can lead to the occurrence of errors in the care provided to inpatients, the search for evidence indicating paths for the adoption of interventions focused on the quality of the care and on the patients' safety, and the low level of knowledge in relation to the phenomenon of interruption in the context of the nursing care in Brazil, this study's objective was to analyze the interruptions experienced by the nursing professionals while undertaking care activities for patients hospitalized in ICU. 

## Method

A quantitative observational transversal study undertaken in two ICUs of a teaching institution located in the Brazilian state of Goiás (GO). 

The study population was made up of all the nursing professionals who undertook nursing activities in the units selected. The observations were made during the morning shifts (from 08:00 to 11:00) and afternoon shifts (from 13:00 to 16:00) in June - August 2014. 

All the nursing professionals were observed individually, once, and simultaneously by two nurses, with the objective of obtaining trust in the data observed. The observers received training regarding data collection (4 hours each) and participated in the pilot study (3 hours each). The degree of agreement between the observers was 94.3%. The discrepant cases were excluded.

For this study, 'interruption' was considered to be any act or attitude whatsoever which interrupted/suspended/broke or diverted the professional's attention from what she was doing, even if temporarily, caused by environmental and/or human factors. 

A semistructured script was used for data collection, validated by two nurses and one specialist in patient safety, with closed questions for obtaining data characterizing the professionals and the environment, followed by a spreadsheet to be filled out with information relating to the interruptions observed: description, the time of the beginning and end of the activity; occurrence, time of beginning and end of the interruption; and source and cause of the interruption. The observers made use of a chronometer, as a means of checking the duration of each activity observed and of each interruption identified. 

The causes of interruption were categorized as described below: 

Related to the patients: Interruptions in which the professional acts in order to qualify her care or that of another professional, seeking or providing information on care practices or the clinical situation of the patient under her care; resolving changes in the clinical situation; providing assistance and giving attention to patients and family members. 

Interpersonal Communication: Interruptions related to communication which is not related to the nursing activity, such as participation in social conversations and parallel conversations. 

Nursing tasks: Interruptions for providing information regarding patients who are not under that nurse's care; entering information in records; adjusting equipment; bureaucratic work. 

Materials: Interruptions for resolving the lack of some or other material while undertaking a procedure, separating and preparing materials for later procedures. 

Movement of people in the environment: interruptions caused by people circulating in, entering or leaving the unit where the care was taking place. 

Telephones: Interruptions for answering cell phones or the unit's telephone. 

Television: Interruptions for paying attention to a television program. 

Alarms: Interruptions caused by the noise from the alarms of the medical-hospital equipment such as infusion pumps, cardiac monitors etc. 

People: Interruptions caused by the professional's distraction, without observable external causes, such as looking away. 

Noises: Interruptions caused by sounds emitted in the environment, such as doors closing, items being dropped, etc. 

 The prevalence of the activities with interruptions was calculated by dividing the number of activities with interruptions by the total number of activities with and without interruptions, multiplied by 100. The Mann-Whitney U test was undertaken to ascertain the difference in the duration of the activities with and without interruptions. The correlation coefficient of the duration of the time spent on the activity and the number of interruptions was ascertained based on Spearman's correlation. 

In order to ascertain statistical differences between the number of interruptions by professional category, the Kruskal Willis test was undertaken, with confidence intervals of 95%. The associations which obtained a value of p<0.05 were considered to be statistically significant. 

The project was submitted to, and approved by, the Ethics Committee of the Teaching Hospital of the Federal University of Goiás, under Opinion N. 556.432/2014. The professionals' inclusion took place subsequent to their signing the Terms of Free and Informed Consent (TFIC). 

## Results

A total of 33 professionals participated in the study, equivalent to 75% of all of the professionals, with 18 (54.55%) nursing technicians, eight (24.24%) nurses and seven nursing residents (21.21%). 

During 99 hours, 739 activities undertaken by the nursing professionals were observed, of which 346 (46.82%) experienced at least one interruption. In all, 778 interruptions were observed, making up a total of 7.85 interruptions per hour, that is, one interruption every 7.64 minutes. The prevalence of the interrupted activities was 46.82%, with a confidence interval of 43.24-50.49. The mean number of interruptions per activity was 1.05, with a confidence interval of 0.91-1.20.

All the professionals observed experienced interruptions. Although the nursing technicians undertake the highest number of activities, this was the category which presented the lowest proportion of interruptions per activity, as shown by Table 1. 

**Table 1:** Distribution of the number of activities, number of interruptions, and proportion of interruptions per activity, by professional category. Goiânia, GO, Brazil, 2014

Professional category	Activities	Interruptions	Proportion of interruptions by activity
Nurse	169	218	1.29
Nursing resident	127	189	1.49
Nursing technician	443	371	0.84
Total	739	778	

On average, the interruptions corresponded to 11.08% of the nurses' worktime; to 9.09% of the time of the nursing residents and to 8.81% of the time of the nursing technicians. Generally speaking, on average, the interruptions corresponded to 9.42% of the nursing professionals' worktime. There was no statistically significant difference in the number of interruptions by professional category (p=0.139).

The interrupted activities had longer durations than those which were not interrupted. The median of the interrupted activities was three minutes (minimum=0.5 minutes; maximum=43 minutes), while those which were not interrupted had a median of one minute (minimum=0.5 minutes; maximum=22 minutes). The correlation coefficient of the duration of the length of the activity and of the number of interruptions, according to the Spearman correlation, was of 0.590 and p=0.000, indicating that they are directly proportional. 

It is emphasized that, in relation to the duration of the interruptions, 584 (75.06%) lasted less than one minute, 158 (20.31%) lasted longer than one minute, 32 (4.11%) lasted from two to five minutes, and four (0.51%) from six to 15 minutes. 

Of all the interruptions observed, 449 (57.71%) led to the interruption of the activity undertaken, and in 329 (42.29%) activities, the professionals continued with what they were doing, in spite of their attention being diverted. 

The analysis of the activities undertaken by the professionals made it possible to construct three categories: of direct patient care, indirect patient care, and administrative activity. The activities observed, with and without interruption, are described in Table 2. 

**Table 2:** Distribution of the activities undertaken by the nursing staff, according to the occurrence or not of interruption, by type of activity. Goiânia, GO, Brazil, 2014

Activities undertaken by the nursing staff	Interruption			Total of activities	
	Yes		No		
	N	%	N	%	
Indirect patient care					
Annotation/Nursing records	82	67.77	39	32.23	121
Handwashing	63	37.50	105	62.50	168
Preparation of materials for procedure	24	68.57	11	31.43	35
Preparation of medications	13	50.00	13	50.00	26
Help with the procedure	6	37.50	10	62.50	16
Communication with another professional	2	14.29	12	85.71	14
Disinfection of equipment	2	16.67	10	83.33	12
Setting up ventilator circuit	2	40.00	3	60.00	5
Discarding the patient's physiological eliminations	1	33.33	2	66.67	3
Checking tests	1	100	0	0	1
Organizing patient transport	0	0	3	100	3
Preparation of cushioning devices for reducing pressure	0	0	1	100	1
Subtotal	196		209		405
Direct patient care					
Administration of medications	32	41.03	46	58.97	78
Applying dressings	24	88.89	3	11.11	27
Assessment of the patient	14	63.64	8	36.36	22
Bedbathing	11	91.67	1	8.33	12
Checking vital signs	10	27.78	26	72.22	36
Cardiac Monitoring	8	57.14	6	42.86	14
Changing position	5	27.78	13	72.22	18
Assistance with oral diet	5	35.71	9	64.29	14
Changing of items attaching endotracheal tube to patient	4	66.67	2	33.33	6
Bed-making	4	80.00	1	20.00	5
Adjusting the patient's bed	3	27.27	8	72.73	11
Collecting material	3	42.86	4	57.14	7
Endotracheal aspiration	3	42.86	4	57.14	7
Aspiration/oral and nasal hygiene	2	40.00	3	60.00	5
Administration of enteral diet	2	25.00	6	75.00	8
Intimate cleaning of the patient	2	50.00	2	50.00	4
Communication with patient/family	1	20.00	4	80.00	5
Preparation of body after death	1	100	0	0	1
Nasogastric intubation	1	100	0	0	1
Admitting the patient	1	100	0	0	1
Installation of parenteral nutrition	1	100	0	0	1
Restraining the patient	1	100	0	0	1
Oral hygiene	1	100	0	0	1
Undertaking electrocardiogram	1	100	0	0	1
Inserting Peripherally Inserted Central Catheter	1	100	0	0	1
Preparation of food	1	100	0	0	1
Subtotal	142		146		288
Administrative activity					
Telephone - work use	7	15.91	37	84.09	44
Computer - work use	1	50.00	1	50.00	2
Subtotal	8		38		46
Total	346		393		739

In the above table it is possible to identify that the activities which experienced the highest number of interruptions were those related to indirect care (196/ 56.65%), followed by those of direct care (142/41.04%) and administrative activities (8/2.32%). Among the activities of indirect care, emphasis is placed on the "annotations and nursing records", with 82 interruptions; in those of direct care, the "administration of medications", and in administrative activity, the "use of the telephone". 

The study also made it possible to identify the sources of interruption, as shown in Table 3. 

**Table 3:** Distribution of the sources of interruption of nursing activities. Goiânia, GO, Brazil, 2014

Sources of interruptions	N	%
External Source - Human Being		
Health professionals		
Nursing technician	181	22.79%
Nurse	64	8.06%
Doctor	36	4.53%
Nursing resident	33	4.16%
House officer	32	4.03%
Other health professionals	27	3.40%
Bursary-funded student nurse	21	2.64%
Laboratory staff	10	1.26%
Medical students	1	0.13%
Subtotal	405	51.00%
Others		
Professionals from other areas	25	3.15%
Patients	22	2.77%
Family members	4	0.50%
Subtotal	51	6.42%
External Source - Environment		
Movement of persons in the environment	32	4.03%
Television	19	2.39%
Equipment (alarms, monitors)	16	2.02%
Telephone (landline)	13	1.64%
Cellphone	3	0.38%
Subtotal	83	10.45%
Internal source		
Self-interruption*	255	32.12%
Subtotal	255	32.12%
Total	794	100%

*Self-interruption: the professional herself causes the interruption of her activity, without the intervention of another person^11^.

It is highlighted that it was possible to identify a total of 794 sources of interruptions, given that some activities were interrupted by more than one source simultaneously. 

The causes of the interruptions were brought together into 11 categories, as presented in Table 4. 

**Table 4:** Distribution of the causes of the interruptions of the nursing activities. Goiânia, GO, Brazil, 2014

Causes of the interruptions	N	%
Related to the patients	270	34.70
Interpersonal communication	206	26.47
Nursing tasks	124	15.94
Materials	56	7.19
Movement of persons in the environment	43	5.52
Telephone equipment	21	2.70
Television	19	2.44
Alarms	15	1.94
People	15	1.92
Noises	7	0.90
Others	2	0.25
Total	778	100.00

## Discussion

The nursing staff experienced a large number of interruptions, on average, 7.85 per hour, many of which were unnecessary, and most of which originated from the health professionals themselves, with emphasis on the nursing technicians, mainly for causes related to the patients under their care and to establish interpersonal communication, such as social conversations. 

Interruptions of the nursing activities have been evidenced in other studies, varying from 0.3 to 13.9 interruptions per hour^2^. A study undertaken in Germany, during the observation of nurses from surgical care units and ICU, identified one interruption every six minutes^12^. In one teaching hospital in the city of São Paulo, the ICU nurses experienced one interruption every eight minutes^11^. In one cardiovascular ICU in a Canadian teaching hospital, an average of 19.7 interruptions per hour was ascertained^9^. 

The positive correlation between the duration of an activity and the number of interruptions suggests that activities which require more time from the professional must be planned and receive interventions in order to minimize unnecessary interruptions. 

The interruptions may cause cognitive errors, including failures in attention, memory or perception^13^, affecting concentration and contributing to the human being forgetting what she was doing, increasing the probability of committing errors^2^ ^-^ ^3^ ^,^ ^14^. The consequence of these failures causes delays in the care, loss of concentration, incomplete work, the omission of the care, and an increase in the risk of errors and exposure of the patient to errors^12^.

The interruptions may also cause negative emotional responses for some professionals, causing them to feel frustrated, stressed and demotivated due to having been interrupted^3^ ^,^ ^15^.

In relation to the time taken up by the interruption and to the disruption/interruption of the activity, it was observed that short interruptions, such as those found in the majority of the cases in this study, cause the professionals to remember what they were doing and to restart their activities with less difficulty, as they reduce the cognitive effort^10^, although they continue to be potential risk factors, considering that each human being reacts differently, at different times, when interrupted. 

In the present study, in 42.29% of the activities interrupted, the professionals had their attention divided between continuing the activity and paying attention to the interruption. This may be a problem for the quality of the healthcare, bearing in mind that in undertaking processes of risk, the professionals' level of attention must be raised and any interruption may lead to the occurrence of errors^8^. The study undertaken in ICU indicated that in 6.6% of the interrupted activities, the professionals had their attention diverted, causing them not to return to the task or to have had their return to the task impeded by a further interruption or due to change in the context of the care^10^.

Regarding the type of activity interrupted, the majority were of indirect patient care, with emphasis on annotations and nursing records. This activity was also among those interrupted most in emergency units of two Swedish hospitals (27.0%)^16^, as well as in clinical and surgical units of a teaching hospital in Toronto, Canada (29.3%)^17^.

When interrupted during documentation, the professional may forget to record information which is essential for patient care and for continuity of the patient's care. It is worth remembering that the records indicate the quality of the care which is being provided, and that the patient record is the main means of communication within the health team, as well as being a legal instrument and contributing to the auditing of the nursing for teaching and research^18^.

In relation to the interruptions which occurred during handwashing, these may be concerning, as they can lead to the omitting or incorrect undertaking of some steps of this technique, impeding the correct hygienization of the entire hands and causing failure to remove the microbiota colonizing the hand, placing the patient's safety at risk^19^.

Frequent interruptions were also found, in this study, during the administration of medications. In ICU, the administration of multiple medications occurs intravenously, many of which are potentially dangerous, this being a high-risk process. In cases of lack of attention on the part of the professional, errors may occur and cause severe harm to the patients^5^ ^,^ ^20^. Studies have indicated that approximately 50% of medication errors take place as a result of distractions caused by interruptions^5^.

Nearly half of the interruptions were caused by the health professionals themselves, as shown in previous studies^6^ ^,^ ^14^ ^,^ ^21^. Unfortunately, health professionals have not yet been made properly aware of the impact which interruptions can have on the quality and safety of the care which is being provided^22^ ^-^ ^23^. As a result, it is necessary to adopt strategies for raising awareness and educating the professionals regarding when interruptions should or should not be avoided. 

In addition to the main source of interruption having been the professionals themselves, attention is called in this study to the high number of self-interruptions which took place during the undertaking of nursing activities, as emphasized in previous studies ^6^ ^,^ ^12^ ^,^ ^24^. This type of interruption can be avoided through better planning of the practice and through raising awareness that while undertaking work activities, the resolution of personal needs can wait^11^.

The self-interruptions due to lack of materials for concluding procedures demonstrate a lack of planning on the part of the professional. One study ascertained that professionals spent approximately 0.6 minutes per hour on interruptions due to lack of supplies, which corresponds to 1% of the work shift^11^, reflected in delays in the care. The use of checklists can be an important tool used for minimizing situations of forgetfulness in preparing materials for procedures. 

Regarding the causes of the interruptions, the fact that the most frequent was that related to the patient, in which the professional acted qualifying her care or that of another professional, suggests that some interruptions have a positive impact on the care. At some points, such as in obtaining information on the care of the patient, or for stopping somebody from going ahead with an unsafe or flawed act, increasing the precision of actions, or improving the patient's condition, the interruptions may be well regarded^12^ ^,^ ^17^. 

Nevertheless, this study also evidenced that interpersonal communication was the second most common cause of interruption. The professionals either interrupted or had their care activities interrupted in order to deal with topics of personal interest, outside the context of what they were involved in. This is a type of interruption which must be deferred, due to the consequences which it can bring to the quality of the care. 

In order to prevent this type of situation, some measures have been proposed, such as the use of colored tops during the preparation and administration of medications, as a signal that these people are not to be disturbed during this activity, or preparation of medications in cubicles^20^. Another approach for reducing interruptions is to make specific areas available for undertaking complex and risky activities, such as preparation of medications, where interruption is not permitted or is limited to urgent communications^25^.

In this context, the need is clear for nurses to analyze the circumstances in which interruptions take place, seeking to avoid those which do not aim for the quality of the care and which can be deferred, thus avoiding the creation of situations which compromise patient safety. 

## Conclusion

Interruptions regularly take place during activities undertaken by nurses who work in ICU. The majority of interruptions took place while nurses were recording their actions or washing hands, and were caused mainly by the health professionals themselves. 

Many interruptions took place with the aim of qualifying the care provided to the patients; however, another large proportion took place for establishing conversations which were not related to the care, and which could be risk factors for reducing the professional's performance and for giving rise to errors, compromising patient safety. 

The present study makes it possible to understand the occurrence of interruptions in nursing practice environments, indicating that interventions which aim to reduce the risk of compromising the professional's performance and to increase patient safety must be put into effect. It also provides valuable data for future studies and support for other researchers, as well as for health managers, indicating situations of risk for care errors and areas where improvements could be adopted. 

As with any observational study, this presented limitations regarding the risk of the presence of the observers having influenced the professionals' behavior. Although the nursing team investigated knew that it was being observed, the other professionals and family members did not; it is therefore believed that this fact may mitigate the risk of the results having been influenced by the method of data collection. It is also considered to be a limitation that the study was undertaken in the ICU, restricting the field to a hospital institution and to a specific population, limiting the results found to similar groups; likewise, the observations were not undertaken at nighttime, when there may be different frequencies and patterns of interruptions. 

Future investigations must be undertaken with a broader population, with varying scenarios, and directed towards the analysis of specific care activities, aiming to extend knowledge regarding the impact of interruptions on the professionals' performance and, consequently, on the quality of the care and on patient safety, and allowing a greater understanding of the phenomenon studied. 

## References

[1] Kohn L, Corrigan J, Donaldson MS (2000). To Err Is Human: Building a Safer Health System.

[2] Hopkinson SG, Jennings BM (2013). Interruptions during nurses' work a state-of-the-science review. Research in Nursing & Health.

[3] Rivera AJ (2014). A socio-technical systems approach to studying interruptions Understanding the interrupter's perspective. Appl Ergonom.

[4] Pereira BMT, Pereira AMT, Correia CS, Marttos AC, Fiorelli RKA, Fraga GP (2011). Interrupções e distrações na sala de cirurgia do trauma: entendendo a ameaça do erro humano. Rev Col Bras Cir.

[5] Feil M (2013). Distractions and Their Impact on Patient Safety. Pennsylvania Patient Safety Advisory.

[6] Institute for Safe Medication Practices (2012). Side tracks on the safety express. Interruptions lead to errors and unfinished... Wait, what was I doing?.

[7] Westbrook JI, Coiera E, Dunsmuir WTM, Brown BM, Kelk N, Paoloni R (2010). The impact of interruptions on clinical task completion. Qual Saf Health Care.

[8] Westbrook JI, Woods A, Rob MI, Dunsmuir WTM, Day RO (2010). Association of Interruptions With an Increased Risk and Severity of Medication Administration Errors. Arch Intern Med.

[9] Sasangohar F, Donmez B, Easty A, Storey H, Trbovich P (2014). Interruptions experienced by cardiovascular intensive care unit nurses An observational study. J Crit Care;.

[10] Grundgeiger T, Sanderson P, MacDougall HG, Venkatesh B (2010). Interruption Management in the Intensive Care Unit Predicting Resumption Times and Assessing Distributed Support. J Exp Psychol Appl.

[11] Monteiro C (2013). Interrupções de atividades realizadas por enfermeiros de um hospital universitário: implicações para a segurança do paciente.

[12] Kalisch BJ, Aebersold M (2010). Interruptions and Multitasking in Nursing Care. Jt Comm J Qual Patient Saf.

[13] Antoniadis S, Passauer-Baierl S, Baschnegger H, Weig Ml (2014). Identification and interference of intraoperative distractions and interruptions in operating rooms. J Surgical Res.

[14] Sevdalis N, Undre S, McDermott J, Giddie J, Diner L, Smith G (2014). Impact of intraoperative distractions on patient safety a prospective descriptive study using validated instruments. Wld J Surg.

[15] Sørensen EE, Brahe L (2014). Interruptions in clinical nursing practice. J Clin Nurs.

[16] Berg LM, Källberg AS, Göransson K, Östergren J, Florin J, Ehrenberg A (2013). Interruptions in emergency department work an observational and interview study. Qual Saf Health Care.

[17] McGillis Hall L, Pedersen C, Fairley L (2010). Losing the Moment Understanding Interruptions to Nurses' Work. JONA.

[18] Maziero VG, Vannuchi MTO, Haddad MCL, Vituri DW, Tada CN (2013). Qualidade dos registros dos controles de enfermagem em um hospital universitário. Rev Min Enferm.

[19] World Health Organization (2009). World alliance for safer health care. Guidelines on hand hygiene in health care. First global patient safety challenge clean care is safer care.

[20] Prakash V, Koczmara C, Savage P, Trip K, Stewart J, McCurdie T (2014). Mitigating errors caused by interruptions during medication verification and administration interventions in a simulated ambulatory chemotherapy setting. BMJ Qual Saf.

[21] Redding DA, Robinson S (2009). Interruptions and Geographic Challenges to Nurses Cognitive Workload. J Nurs Care Qual.

[22] Raban MZ, Westbrook JI (2013). Are interventions to reduce interruptions and errors during medication administration effective : a systematic review. BMJ Qual Saf.

[23] Raban MZ, Lehnbom EC, Westbrook JI (2013). Interventions to reduce interruptions during medication preparation and administration.. Australian Commission on Safety and Quality in Health Care, University of New South Wales.

[24] Brixey JJ, Tang Z, Robinson DJ, Johnson CW, Johnson TR, Turley JP (2008). Interruptions in a level one trauma center A case study. Int J Med Inform.

[25] Anthony K, Wiencek C, Bauer C, Daly B, Anthony MK (2010). No Interruptions Please Impact of a No Interruption Zone on Medication Safety in Intensive Care Units. Crit Care Nurse.

